# MENTAL HEALTH VULNERABILITIES AND STRENGTHS OF OLDER WOMEN WITH HIV DURING THE HUMANITARIAN CRISIS IN UKRAINE

**DOI:** 10.1093/geroni/igad104.0449

**Published:** 2023-12-21

**Authors:** Julia Rozanova, Sheela Shenoi, Oleksandr Zeziulin, Katherine Rich, Dan Bromberg, Tetiana Kiriazova, Alexandra Deac, Valerie Earnshaw

**Affiliations:** Yale University, New Haven, Connecticut, United States; Yale School of Medicine, New Haven, Connecticut, United States; Ukrainian Institute of Public Health Policy, Kyiv, Kyyiv, Ukraine; Harvard Medical School, Boston, Massachusetts, United States; Yale School of Public Health, New Haven, Connecticut, United States; Ukrainian Institute of Public Health Policy, Kyiv, Kyyiv, Ukraine; King’s College London, London, England, United Kingdom; University of Delaware, Newark, Delaware, United States

## Abstract

Humanitarian crises impact mental health, yet data lacks among older individuals. We explored how Covid-19 and the Russian invasion has impacted older people with HIV (OPWH, ≥50 years) in Ukraine. We surveyed a longitudinal cohort of 123 OPWH (50% female) during four periods: April-June 2020 (Wave 1), December 2020-February 2021 (Wave 2), December 2021–February 2022 (Wave 3), and July–September 2022 (Wave 4). The primary outcomes were depressive (PHQ9>5) and anxiety symptoms (GAD7>5). Factors associated with these symptoms were assessed using a mixed effects logistic regression model that included gender, time since HIV diagnosis, history of a substance use disorder (SUD), history of another chronic condition, HIV disclosure, living alone, and social support. Logistical regression controlling for SUD assessed factors associated with resilience (BRS). During Wave 4, OPWH were offered free psychological counseling through our project. Across all Waves, women were more likely than men to have mild to severe anxiety and depressive symptoms, but also to have higher resilience, and to accept psychological counseling during the war. Comorbid SUD and other chronic conditions increased likelihood of anxiety, and longer time since HIV diagnosis increased likelihood of depressive symptoms. Unlike women, men showed distress discussing “Social Support” topic during the war. OPWH with SUD had higher resilience across all waves. While female OPWH had more depressive and anxiety symptoms during the crises they have higher resilience, social support, and help acceptance, suggesting bespoke approaches are needed to assist male and female OPWH in humanitarian settings.

